# 

**DOI:** 10.1192/bjb.2020.135

**Published:** 2021-06

**Authors:** Amit D. Mistry

**Affiliations:** Chair of the Royal College of Psychiatrists Sport and Exercise Special Interest Group, Oxford Health NHS Foundation Trust, UK. Email: amist85@gmail.com

The ‘*Cognitive Sports Therapy Manual’* is a 120-page book centred on holistic, fundamental concepts, such as ‘mind, body, breath’, for supporting mental health. It is based on 12-week practical courses and support groups (available at www.cognitivesportstherapy.com), designed by a founding Multi Disciplinary Team composed of psychiatric, general practice, yoga and exercise professionals. It includes the use of non-medical jargon and practical tools, such as a gratitude journaling, screening tools, care planning, worksheets and calendar logs, for tracking personal progress related to ‘mind, body and breath’ exercises.

This type of manual is timely, particularly given the emerging robust evidence base for the therapeutic role of ‘lifestyle psychiatry’ (e.g. exercise, nutrition, sleep, stress management and adverse health behaviours) within severe mental illness. The back of the manual makes reference to some of these key papers and texts.

Within front-line psychiatry work, the use of some of these proposed, alternative therapeutic methods is well aligned with the preventative direction of the NHS Long Term Plan. This may prompt traditional Multi Disciplinary Teams to include professionals who can optimise such lifestyle factors (e.g. physiotherapists), particularly when patients with severe mental illness cite lack of staff support as a major barrier to physical activity engagement.

Despite idealism toward holistic interventions, we also have evidence from pragmatic trials led by Gaughran et al (https://doi.org/10.1186/s12888-017-1571-0), demonstrating the challenges of embedding positive lifestyle factors for severe mental illness. Therefore, this type of manualised approach may be more suited as a well-being strategy for those with higher levels of motivation. Further, I believe that the book should have included a screening function or content related to the risks of over-reliance on exercise as a coping mechanism, as we know this can result in exercise addiction and associated dysfunctional eating behaviours.

In summary, I enjoyed reading this manual and knowing that there are professional initiatives exploring holistic, lifestyle factor optimisation that can benefit individuals across the mental health spectrum.

